# Looking Ahead: Advancing Measurement and Analysis of the Block Design Test Using Technology and Artificial Intelligence

**DOI:** 10.3390/jintelligence12060053

**Published:** 2024-05-22

**Authors:** Kiley McKee, Danielle Rothschild, Stephanie Ruth Young, David H. Uttal

**Affiliations:** 1 Department of Psychology, Northwestern University, Evanston, IL 60208, USA; 2 Department of Medical Social Sciences, Northwestern University Feinberg School of Medicine, Chicago, IL 60611, USA

**Keywords:** assessment, measurement, technology, artificial intelligence, block design test

## Abstract

The block design test (BDT) has been used for over a century in research and clinical contexts as a measure of spatial cognition, both as a singular ability and as part of more comprehensive intelligence assessment. Traditionally, the BDT has been scored using methods that do not reflect the full potential of individual differences that could be measured by the test. Recent advancements in technology, including eye-tracking, embedded sensor systems, and artificial intelligence, have provided new opportunities to measure and analyze data from the BDT. In this methodological review, we outline the information that BDT can assess, review several recent advancements in measurement and analytic methods, discuss potential future uses of these methods, and advocate for further research using these methods.

## 1. Introduction

Despite exponential advancements in technology in recent years, many cognitive and intelligence tests are still administered and scored using traditional paper-and-pencil methods. Even spatial tests, which measure one’s ability to reason about, evaluate, and solve problems dealing with physical spaces and objects or representations of such stimuli, are still administered with paper and pencil. However, a few spatial tests use physical manipulatives, particularly blocks, to measure spatial assembly and construction abilities. Thus, these physical spatial tests have unique affordances for capturing spatial reasoning through action (Shelton et al. 2022). One of the most used block-based spatial tests is the block design test (BDT; Kohs 1920; Wechsler 1939, 2008), a test of spatial visualization that requires spatial assembly, though there have been several other tests that utilize cube-like blocks and building blocks, like LEGO blocks (Casey et al. 2008; Cortesa et al. 2017; Verdine et al. 2014).

The BDT and related tasks are a rich source of information on spatial thinking. For example, the BDT requires examinees to evaluate the design and the sides of the blocks, rotate the blocks such that the correct side is facing up and in the correct orientation, plan for the next block placement, and monitor their progress throughout the task. The cognitive complexity of the BDT reflects real-world examples of spatial assembly tasks, such as assembling furniture or working with mechanical systems.

Traditionally, the primary BDT score is based on the final accuracy of the block placements and the time required (Wechsler 2008), but relying solely on this standardized score sacrifices important information about the building process (Joy et al. 2001). Given the action-by-action nature of the task, it is possible to evaluate not just the final product but *how* individuals obtain the final product. Existing supplementary process scores include analyzing performance without consideration of time bonuses, partial accuracy, and certain types of errors. However, even these supplemental scoring systems do not capture information about each step taken to complete the design (Lichtenberger and Kaufman 2009; Kaplan 1991; Ryan et al. 2013; White and Rose 1997). Process variables such as block placements, time information, and looking behavior, could be particularly useful for better understanding individual differences in spatial cognition (Salthouse 1987; Shah and Frith 1993), as well as in a clinical diagnostic setting (Kaplan 1991; White and Rose 1997). Thus, nuanced scoring systems that record more detail would be highly valuable, but most clinicians and researchers cannot measure and record more detailed information when administering and scoring tests in real time. Modern advancements in technology and artificial intelligence allow for a more detailed examination of the cognitive processes used throughout the test.

In this methodological and theoretical review, we provide an overview of the history of the BDT, reflect on the test’s usefulness in cognitive research, clinical, and educational settings, describe advancements in technologies related to the BDT, and examine future directions.

## 2. History of the Block Design Test

Kohs introduced the BDT as a non-verbal performance test (Kohs 1920). Participants were asked to use multi-colored blocks to construct three-dimensional copies of visual designs. The designs increased in difficulty as the test progressed, starting with symmetrical patterns that required fewer blocks to more complex patterns that required many blocks. There were 17 different designs, ranging from 4-block to 16-block designs. Kohs’ scoring system included final accuracy, total completion time, and the number of moves taken to complete the design. In this context, “move” is defined by the placement of a block. Increased time and number of moves taken to complete the design contributed to lower scores while speed and accuracy led to higher scores.

Kohs introduced the test as a step away from the focus on faculty psychology, which centers on the idea of separating the mind into different faculties, like judgement and attention (Brooks 1976); instead of being a test for a specific, separate cognitive process, the BDT requires multiple processes (Kohs 1920). Kohs compartmentalized the required processes to include the ability to understand and maintain the goal design, placing blocks so their combination works towards completing the goals, and evaluating the placed blocks and comparing them to the goal. He proposed that the BDT was a measure of general intelligence as it required the combination of several distinct cognitive processes. Particularly, Kohs supported the notion that synthesis and combination were particularly important aspects of human intelligence (Kohs 1923). In his standardization of the test, he highlighted its correlation with the Binet scales, furthering the claim that his test was a comprehensive measure of non-verbal intelligence.

As psychologists and psychiatrists started testing patients with the BDT in their practices, there became an increasing demand for easier-to-administer versions of the BDT, particularly as the test was initially supplemental to other more established tests (Wile and Davis 1930). For example, Hutt introduced a new scoring method that removed the counting of the number of moves an individual made (Hutt 1930, 1932). In the 1930s, Wechsler further adapted Kohs’s BDT for his Wechsler–Bellevue Intelligence Scales (WBIS) (Wechsler 1939). Most of the changes in this adaptation were intended to shorten and simplify the test for use as part of a larger battery of tests. Wechsler decreased both the number of items and the colors used. Further, he claimed that the BDT was the single best predictive scale from the entire battery and that it correlated highly with the total test score and other individual tests, including verbal measures. By 1955, an updated form of the WBIS was introduced, named the Wechsler Adult Intelligence Scales (WAIS), which soon surpassed the Stanford–Binet Intelligence Scales as the most popular and commonly used test of intelligence (Lichtenberger et al. 2006). It is within WAIS that the BDT has most often been administrated, though it has not been limited to this context. In 1949, Wechsler developed the Wechsler Intelligence Scale for Children (WISC), which was modeled after the WAIS but introduced easier items to adapt the existing tests, including the BDT, for children (Littell 1960).

## 3. Usage and Importance of the Block Design Test

In the decades since its creation, the BDT has been used not just for general cognitive testing but also for clinical, educational, and research purposes.

### 3.1. Block Design as a Diagnostic Tool in Clinical Settings

For almost as long as the BDT has existed, clinicians have used it to estimate cognitive abilities in a variety of settings across the lifespan (Hutt 1932). The BDT can also be used to help diagnose ADHD (Antshel et al. 2007), autism spectrum disorder (Stewart et al. 2009), and other developmental disorders in children (Cardillo et al. 2017). At the other end of the lifespan, the BDT has been used in research on aging and cognitive decline (Joung et al. 2021a; Rönnlund and Nilsson 2006) and may help differentiate adult participants with dementia, mild cognitive impairment, and Alzheimer’s Disease, as well as healthy adults (Devanand et al. 1997; Joung et al. 2021b; Yin et al. 2015).

The BDT is particularly useful for assessing non-verbal learning disability (NLD) in clinical contexts. NLD is a developmental disorder characterized by lower visuospatial intelligence and memory and is often assessed by comparing children’s performance on the BDT to other WISC subscales (Mammarella and Cornoldi 2014). Children with NLD tend to perform worse than typically developing children on the BTD, both in terms of accuracy and speed, while performance tends to be within the normal range on verbal measures, such as vocabulary from the WISC (Mammarella et al. 2019; Pelletier et al. 2001; Venneri et al. 2003). Children with NLD may struggle with the BDT because of weaknesses in global processing, which involves reasoning about the relation between the overall structure and sub-structures or individual blocks (Cardillo et al. 2017). In cases of highly organized designs, where different parts of the design are highly cohesive and related, children with NLD, on average, could only arrange about half as many blocks correctly as typically developing children could (Mammarella et al. 2019). In line with this research, the BDT has helped shed light on differences in visuospatial functioning between highly comorbid developmental disorders, such as NLD, autism spectrum disorder, and ADHD (Cardillo et al. 2020).

The BDT is also commonly used in the comprehensive neuropsychological assessment of patients after a traumatic brain injury, both to assess the extent of cognitive sequelae and monitor recovery over time (Goldstein et al. 2010; Hammond et al. 2004; Millis et al. 2001). Individuals with traumatic brain injuries and other neurological disorders that damage spatial processes, such as epilepsy, tend to have more broken configuration errors on the BDT than non-clinical populations (Akshoomoff et al. 1989; Ben-Yishay et al. 1971; Wilde et al. 2000; Zipf-Williams et al. 2000). In a case study of three patients with brain injuries, Toraldo and Shallice (2004) analyzed BDT performance at the individual move level and found that their patients made several different types of errors, including broken configurations, rotations, overestimation of dimensions of certain design aspects, and missing blocks.

Lastly, the BDT can also help clinicians create individualized intervention plans for their patients. For example, information from the BDT can help neuropsychologists create targeted recommendations for patients to better cope with spatial deficits at school and work. Academic accommodations might include specialized mathematics instruction, such as multisensory instruction, and extra time in academic settings (Doty 2019).

### 3.2. The Block Design Test in Research and Educational Assessment

The BDT has been classified as a test of spatial visualization and has been used to predict functional spatial skills for decades (Casey et al. 2008; Fenouillet and Rozencwajg 2015; Grote and Salmon 1986; Linn and Petersen 1985). Groth-Marnat and Teal (2000) found that the BDT was related to everyday spatial abilities, such as arranging furniture and packing boxes, thus providing evidence of the measure’s predictive validity in real-world settings. Block-based construction tasks, such as the BDT, predict achievement and entering careers in STEM fields (Fernández-Méndez et al. 2020; Hsi et al. 1997; Tian et al. 2023; Verdine et al. 2014; Wolfgang et al. 2001); consequently, the BDT is used frequently in educational assessment and research. Comprehensive cognitive assessment identifies patterns of strengths and weakness and supports the creation of individualized education plans (IEPs) (Grigorenko et al. 2020). Cognitive assessments have also been used to identify talented children, and there is growing interest in “spatial giftedness” (Ballard 1984; Hagmann-von Arx et al. 2008; McCoach et al. 2001; Silverman and Gilman 2020; Lakin and Wai 2020; Wiese et al. 1988).

Demonstrations of the role of spatial skills in STEM success have led to a new interest in *spatial training*, which involves attempting to increase spatial skills and, eventually, STEM (usually mathematics) performance (e.g., Hawes et al. 2022; Uttal and Cohen 2012). Spatial abilities are malleable (Uttal et al. 2013); spatial training and experiences can improve spatial abilities (Sorby et al. 2013; Wright et al. 2008), including block design performance (Day et al. 1997; Dirks 1982). In addition, more general spatial experiences, such as block play, are also related to higher block design scores (Casey et al. 2008; Jirout and Newcombe 2015). Thus, the BDT and related block construction tasks may be more than an assessment; they may also potentially be an intervention or teaching tool.

## 4. Moving beyond the Limitations of the BDT

Traditionally, the BDT yields only a single accuracy score and the time required for the construction (Hutt 1930, 1932). This scoring system has been used for almost a century because it is efficient and provides valid diagnostic information for several aspects of intelligence and clinical disorders. Moreover, the scoring system can be taught and learned relatively easily.

However, the single numeric score does not capture all the complexities and individual differences that occur during the BDT (Dunn et al. 2021). Completing the multiple steps of the BDT requires the use of spatial information, executive function, and working memory (Landau and Hoffman 2012). Test-takers must examine and parse the target design, hold elements of the design in their visuospatial working memory while selecting a block, decide which block to select, and properly orient and place the block in their copy (Ballard et al. 1995; Kohs 1920; Shah and Frith 1993). The cognitive demands of this task reflect Kohs’ original intent in designing the BDT purposefully to draw upon multiple cognitive functions (Kohs 1920). Further, there are no restrictions on *how* test-takers complete the task. Individuals may differ in how they segment the target design, which blocks they select, and the placement of blocks into their copy, resulting in different possible strategies (Rozencwajg 1991; Salthouse 1987; Shah and Frith 1993).

The traditional scoring system does not reflect many of these individual differences, and consequently, many potential insights into cognitive processes are overlooked. Addressing these limitations could be transformative; we know from other cognitive tasks that a step-by-step analysis of construction processes and errors can provide much greater insight into the underlying cognitive factors involved in the task. For example, modeling of the Tower of Hanoi, a problem-solving test in which individuals must rearrange rings on pegs, has shown that subgoals that split the task into smaller components may play an important role in how individuals approach the task (Donnarumma et al. 2016; Kotovsky et al. 1985). The physical properties of the Tower of Hanoi make it easier for researchers to identify and model solution strategies. Likewise, we suggest that the physical nature of the BDT allows for more detailed examination of the cognitive sub-processes involved than other spatial tasks do.

To realize the potential of the BDT to provide insights into cognitive processes, we must be able to record details of the test that extend beyond the limits of the traditional scoring method. One possibility is to videotape a participant as they construct the designs and to watch the videos (often many times) to gather the necessary information. However, manually reviewing video recordings is both time-consuming and tedious, especially in the context of large research studies. Moreover, extracting data from videos also requires creating reliable and analyzable coding schemes, which can be very challenging. Such challenges have limited the level of detail and depth of analyses of BDT responses. Although many researchers have attempted to advance understanding and classification of the cognitive processes that individuals use in completing the BDT, these efforts have been limited in scope. For example, Joy et al. (2001) recorded block placements during BDT constructions and described various kinds of errors and block placements that participants made. However, while reporting on the presence and frequency of certain types of errors can offer some insight into individual differences, it does not capture the entirety of possible errors and does not allow for easy comparison of total block placement sequences. Furthermore, this kind of hand coding also leaves out certain variables, such as time for each individual block placement. Thus, while there have been several researchers who have studied various aspects of the BDT building processes, researchers have not been able to provide a complete description or categorization of these processes (Dunn et al. 2021). Fortunately, with the rise of new technologies and artificial intelligence, there are several emerging methods that can greatly increase the amount of available information that can be captured during a BDT construction and present new ways to analyze such data (Cha et al. 2018, 2020; Lee et al. 2016).

## 5. Technological Advancements for Capturing BDT Actions

Advances in technology now give researchers and clinicians multiple ways to take advantage of the physical nature of the BDT. Here, we discuss how new technologies can capture important behavioral elements of the BDT, such as eye gaze and the block placing sequence. Additionally, we discuss the potential uses of artificial intelligence for analyzing the detailed information obtained from the new methods.

## 6. Measuring Gaze and Fixation Behavior during the BDT

Eye-tracking has a long history in the study of cognition, and major advancements have recently been made in eye-tracking measures (Mele and Federici 2012). We can learn a lot about individuals’ strategies on the BDT by analyzing which test components they look at, how long they look, and the sequence of their gazes (Fenouillet and Rozencwajg 2015). For example, the sequence of when participants look at the target design, the bank of blocks, and the copy area can shed light on individual strategies on spatial construction tasks (Hayhoe et al. 1998). Further, the number of times that individuals look back to the target design can shed light on their working memory—that is, how well they can maintain the mental image of the design while selecting and placing blocks (Ballard et al. 1995). As early as the 1990s, researchers begin to use gaze patterns to classify participants’ strategies on BDT (Rozencwajg 1991). However, these methods were limited in what information they could reveal or record. For example, while eye-tracking can determine if an individual is looking at the target design, more precise equipment is required to determine exactly where in the design the individual is looking or what block face they are looking at when looking to pick up a block from the bank.

Modern eye-tracking techniques implement multiple cameras; an overhead camera can record what the participant is doing, while corneal imaging cameras can identify exactly where the participant is looking (e.g., Besari et al. 2023; Cha et al. 2020; Shigenaga and Nagamune 2022). For the BDT in particular, corneal imaging is the preferred method by which to measure gaze as participants need a wide space to recreate the designs, and a typical monitor-mounted eye tracker or head-mounted gaze tracker often misses eye movements (Cha et al. 2020). Corneal imaging is optimal as it is easy to calibrate and does not require participants to wear unwieldy equipment. It also allows researchers to accurately time how long a participant is looking at a certain area (e.g., the target design, block bank, or construction area).

## 7. The Importance of Individual Block Placements

Despite the challenges in recording detailed behaviors by hand, several studies have contributed to greater understanding by focusing on specific process variables, such as starting position or contiguous block placements. Recently, Dunn et al. (2021) conducted a literature review of these extra variables that have been examined in the BDT. Here, we are particularly interested in block placements and the sequence of block placements.

### 7.1. Sequence of Block Placements 

**Figure 1:**
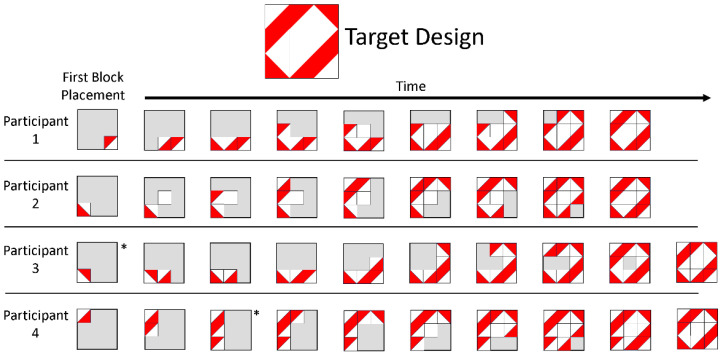
Example of building sequences. This figure shows four potential sequences on block placements from four individuals recreating the target design. States of blocks with an * indicate that an erroneous block placement was made. Erroneous block placement in this sense refers to a block placed that does not match the target design.

The sequence of block placements is perhaps the greatest source of individual differences in the BDT (Jones and Torgesen 1981; Rozencwajg and Corroyer 2001). For example, one person might place the blocks left-to-right, top-to-bottom, while another might place the blocks starting in the four corners and then filling in bottom-to-top. Both constructions are correct in the traditional scoring, but obviously there are differences in how the two participants reached the final state. Figure 1 shows examples of these differences in building; it illustrates four (hypothetical) participants’ constructions of the same design. Although all participants successfully built the target design, each participant used a different building strategy. For example, Participant 1 went side to side and filled out each row, whereas Participant 2 had a less systematic building pattern.

Mathematically, for a 9-block 3 × 3 design, there are over 362,000 different sequences of block placements where each placed block is correct. For 4 × 4 designs, the number of possible sequences increases to over 479 million. Further, this possible sequence space increases when considering erroneous block placements. Recent research indicates that individuals typically adhere to only a few of the possible spatial assembly strategies, thus reducing the number of observed sequences; however, there are still too many sequences to easily track by hand (Shelton et al. 2022).

There are a few ways that sequences have been characterized. Contiguity refers to whether blocks are placed adjacent to already placed block (Dirks 1982) and is one of the only characterizations that maps the entire assembly process (Dunn et al. 2021). Other characterizations focus on certain parts of the assembly process, such as where individuals start the build or if they reach certain pre-final arrangements of blocks, such as completing the outer ring before the middle or completing rows and columns (Cha et al. 2020; Jones and Torgesen 1981).

### 7.2. Erroneous Block Placements

Erroneous block placements, placing blocks that do not match the target design, complicate recording block placing sequences, but they may also shed new light on individual differences. For example, in Figure 1, Participant 3 and Participant 4 both made an erroneous block placement while building. Participant 3 fixed their mistake after one action, while Participant 4 did not fix their mistake until the end of their building. In particular, erroneous block placements can provide insight into how individuals are thinking about the test or their more general cognition, such as global vs. local processing (Kramer et al. 1996, 1999). There are more severe errors, such as not building in the appropriate shape or orientation or not completing the design, that would be recorded in the traditional scoring method, as these kinds of errors would result in a null score for accuracy (Akshoomoff et al. 1989; Akshoomoff and Stiles 1996; Joy et al. 2001). In clinical settings, these kinds of errors may indicate cognitive impairment either from brain injury or neurological or developmental disorder, such as Williams syndrome (Ben-Yishay et al. 1971; Farran and Jarrold 2003). However, the traditional scoring method does not capture errors if they are corrected before the participant is finished completing the design, except for increasing the time taken. Typically, adults may make more subtle errors, such as mistakenly rotating one block, and tend to fix their errors before finishing the task (Joy et al. 2001), whereas typically developing children likely make certain types of errors based on the types of designs or strategies used (Akshoomoff and Stiles 1996). Such errors would not be captured by the traditional scoring method but could partially be captured by recording the number of moves taken.

## 8. Computerized Versions of the Block Design Task

Advancements in technology have allowed for the BDT to be administered in new ways that capture these important behavioral details. These advancements are the result of work in several disciplines and have gone beyond the traditional administration of physical blocks. There are now entirely computerized versions of the BDT, which offer new affordances in how the task is analyzed, particularly because the virtual nature of such administration can allow for the recording of every movement of the blocks.

### 8.1. Virtual Administration

Virtual administration of the BDT allows for instantaneous data collection based on where a participant moves their mouse and clicks. For example, Rozencwajg and Corroyer’s SAMUEL is a software version of the BDT (Rozencwajg and Corroyer 2001; Rozencwajg et al. 2005; Rozencwajg and Fenouillet 2012). SAMUEL splits a computer screen into three areas. The target designs are shown on the left side of the screen, and the right side shows a workspace to reproduce the designs. A third area is located below the other two and contains a bank of virtual blocks for participants to select and drag into the workspace (Rozencwajg and Corroyer 2001). Since the placements are recorded automatically, the experimenter can know immediately when a participant has finished the design. Additionally, SAMUEL records instantaneous solution times, and will therefore be more accurate. The program also records placement order and how often the participants requested the design be shown. SAMUEL has been used with many ages, including 12-year-olds, 17-year-olds, young adults, and middle-aged adults.

More recent investigations have used virtual administration with typically developing and children with learning disabilities. Cardillo et al. (2017) compared the performance of children with symptoms of NLD or dyslexia to typically developing children on virtual tasks based on the BDT. One potential limitation is that children indicated their selections verbally because some children were unable to use the mouse. Researchers manually entered the response time and choices, resulting in a potentially unreliable response time.

### 8.2. Virtual Reality

An advantage of digital administration of the BDT is that block placements can be recorded easily. However, the presentation of the BDT on a computer or tablet screen is quite different from the standard administration, which includes physical manipulatives, and may affect how people approach the test. Virtual reality (VR) can offer the benefits of computerized administration while also being closer to traditional administration. VR has wide ranging applications, and performance on block-related tasks in VR has been found to relate to visuospatial abilities (Averbukh et al. 2019; Wikström et al. 2020).

Shigenaga and Nagamune (2022) directly compared physical and VR administration of the BDT. For both types of administration, they recorded individuals’ eye and hand movements. Participants took longer to complete the VR version and had more grasping motions. This result can be explained by the need to learn how to grasp objects in VR when one is not physically grabbing anything. As VR technology advances, it may be possible to include haptic feedback that more realistically simulates manipulating physical blocks (Shigenaga and Nagamune 2022).

Other researchers have also used the BDT in virtual environments to see if spatial training in these VR environments extends to real world applications (e.g., Averbukh et al. 2019; Park 2022). In one such application, participants with amnesic mild cognitive impairment completed the BDT and other tasks before and after eight weeks of training in a VR environment where they were asked to navigate themselves and find various locations (Park 2022). The participants in this training group increased significantly more than the control group in terms of their WAIS performance at post-test and had greater improved hippocampal function overall. Although the researchers did not administer the BDT in VR, this study shows that VR spatial training does relate to performance on physical spatial tasks.

## 9. Embedded Sensor Systems

Advancements in virtual administration may eventually support the recording of multiple behavioral processes while an individual completes the BDT. However, the traditional, hand-held version still has some important potential advantages. The tactile feedback gained from using blocks may be important, particularly for children. Thus, it would be useful to develop a system that offers some of the advantages of traditional blocks while conveying the advantages of a computer-based system. In this section, we discuss the development of sophisticated physical block systems that can record and easily analyze the step-by-step actions required. These systems use a more sophisticated data collection system that extends beyond the limits of video recording and subsequent coding.

Researchers can use accelerometers and transmitters that are embedded inside specially engineered blocks to record where each block is placed, the order of block placements, and the time to each block placement (Lee et al. 2016, 2018; McKee et al. 2023). This data allows researchers to classify strategies based on those block placements and actions (Rozencwajg and Corroyer 2001; Cha et al. 2020; McKee et al. 2023). Given that the typical administration of the BDT requires a professional to administer, code, and classify, embedded sensor systems provide a less costly and easier alternative (Jeong et al. 2010), allowing clinicians more time during evaluations to gather more detailed information about the patient, rather than focusing exclusively on the time and performance.

### 9.1. Tangible Geometric Games

One such system is the sensor-integrated geometric blocks (SIG-Blocks) that can be used alongside an interactive graphical user interface where participants can complete tangible geometric games (TaG-Games) and be analyzed computationally without an overhead camera (Jeong et al. 2010). These SIG-Blocks are designed with sensors on each contact surface of the blocks and emit infrared signals to each other. Accelerometer and assembly configurations can be displayed on a researcher’s graphical user interface in real time. While other comparable systems have been designed previously, the SIG-Blocks were the first to be wireless, and they track assembly in addition to orientation among other measurable data (Lee et al. 2016). However, analyses of TaG-Games have mostly focused on the speed of completion and overall correctness (Lee et al. 2016, 2018), which is comparable to the traditional methods.

### 9.2. Smart Cubes

One example of how to use technology like sensor systems to understand how participants complete assembly tasks from a cognitive standpoint is sCubes, or Smart Cubes, which can record the real-time movements and connections of blocks while participants complete the BDT (McKee et al. 2023). The system records the actions an individual takes while completing a design, providing a sequence of intermediate states of block placements. In combination, the series of states represent the path taken by an individual to complete the design, and we can use individual participants’ path diagrams to determine what general patterns are taken to complete each design. Thus far, analyses have shown modal paths and convergent states that are indicative of participants using comparable strategies to complete certain designs in the BDT, and the goals of the project include implementing artificial intelligence and machine learning algorithms to analyze these common paths, likely through machine learning clustering algorithms (McKee et al. 2023).

## 10. Artificial Intelligence and Machine Learning in Measuring and Analyzing the BDT

The use of artificial intelligence, and specifically machine learning, is growing rapidly and is particularly useful for scientific endeavors (Mjolsness and DeCoste 2001). Here, we focus on one kind of artificial intelligence, machine learning. Machine learning consists of computational processes, typically classified as algorithms, that can take input and perform a task on that input without the need for explicitly coded instructions (El Naqa and Murphy 2015). Machine learning has been used to automate parts of scientific research, allowing for increasingly larger datasets to be used and analyzed (Rudin and Wagstaff 2014). The use of machine learning in psychology is still relatively new but offers intriguing possibilities (Dwyer et al. 2018; Orrù et al. 2020; Yarkoni and Westfall 2017). There are several ways in which artificial intelligence can be used in cognitive testing, and experts in the fields of cognitive science, computer science, and engineering are currently pioneering these avenues. Kunda (2019) highlights three specific ways in which artificial intelligence can enhance cognitive testing: (1) behavioral sensing, which allows for better monitoring of an individual’s behaviors during a test; (2) data mining, which enables the recognition of patterns from large datasets; and (3) cognitive modeling, which provides a means to examine computational models of cognition. Here, we consider uses of artificial intelligence and machine learning that are specific to the BDT that generally align with Kunda’s (2019) roadmap for enhancing cognitive testing with artificial intelligence. Furthermore, the advancements in eye-tracking, computer administration, and sensor system technology described above also support these uses of artificial intelligence for measuring and analyzing performance on the BDT. Such advancements in hardware and software allow for more data that can be processed using novel artificial intelligence methods.

### 10.1. Computer Vision

One way machine learning and artificial intelligence can be used to automate the process of measuring the BDT is with *computer vision* (El Naqa and Murphy 2015). Computer vision systems can take pictures or video as an input and automatically detect and record objects and actions taking place. Some researchers have begun developing systems that rely on machine learning, artificial intelligence, and computer vision to parse spatial assembly tasks, such as building with LEGO blocks (Jones et al. 2019). For the BDT specifically, the Laboratory for Artificial Intelligence and Visal Analogical Systems (AIVAS Lab) at Vanderbilt has developed an Automated Block Identification System (ABIS) (Cha et al. 2018, 2020).

ABIS employs both machine learning and computer vision to automatically detect block placements during the BDT from overhead video recordings of the task (Cha et al. 2018). The set-up of the task is modified slightly by the addition of a frame and colored background, and an overhead camera is placed above the build area. The overhead video is then processed automatically by computer vision, specifically to detect and determine where blocks have been placed, their orientation, and their color. This identification process runs through each frame of the video, resulting in a frame-by-frame sequence of block states. Further, when applied to videos from multiple test-takers, this system can produce sequences from each individual that are comparable to each other.

### 10.2. Supervised Machine Learning for Classifying BDT Sequence Data

The usefulness of machine learning and automated systems for BDT does not stop at automating the coding of block sequences. Once researchers have obtained these sequences, they can be analyzed and compared. The AIVAS Lab has also introduced a system for analyzing the output of their computer vision system (Cha et al. 2018). Researchers first identified a few distinct strategies that individuals may use on different designs of the BDT, such as always going row-by-row or starting on the outside then completing the inside. Then, the researchers created samples sets of block sequences that would fall into these different strategy categories. Finally, they created a system that could classify the strategy of real participant block sequences by calculating similarity scores between the obtained sequences outputted from their ABIS computer vision system and the researcher-generated sample sequences to determine the best fitting strategy.

### 10.3. Unsupervised Machine Learning Techniques for BDT Analysis

While supervised machine learning techniques can efficiently automate the process of analyzing BDT data, such systems depend on humans instructing the system on how it should classify different data. For instance, Cha et al. (2018)’s analysis system only classifies sequences based on the strategies pre-determined by the human researchers. However, there are machine learning techniques that do not depend on human knowledge to be able to find patterns within datasets. Specifically, *unsupervised machine learning*, which is a process where the system learns from itself on the input data (El Naqa and Murphy 2015). Such methods can be very useful for researchers as these unsupervised machine learning methods can be used to identify patterns in data that humans cannot detect unassisted, particularly in complex datasets (Eckhardt et al. 2023). Researchers in the medical sciences have recently started to use unsupervised machine learning to find patterns among patients based on genome data (Lopez et al. 2018). Such clustering can be useful as it might bring to light certain similarities or differences between different patients previously unrecognized by the physicians or researchers.

For BDT, unsupervised machine learning introduces an opportunity to find important characteristics of how individuals complete the test that may not have been previously considered. This method is currently under development (McKee et al. 2023). For example, if many individuals are recorded by one of the methods above, such as the sCubes sensor system or ABIS computer vision system described above, the unsupervised machine learning algorithms might be able to detect strategies outside of those that researchers have identified from their own observations. Further, such systems can potentially consider multiple types of information at once. Thus far, we have discussed the analysis of the sequence of block placements, but machine learning is not limited to just examining sequences. For instance, an unsupervised machine learning system may combine block sequence information with time information, such as how much time passes between each block placement. This information could help to shed light on why slower building in the BDT is typically associated with worse performance (Troyer et al. 1994; Back et al. 2022). The system can be set up in such a way as to examine aspects of both the sequence of block placements and the placement time information to find new connections between certain strategies and overall ability at the task. Further, with more and more intricate systems, more variables may be examined, such as eye-tracking data synced with block placements or motor data from hand sensors (Besari et al. 2023).

With all uses of machine learning and artificial intelligence for evaluating the BDT, the systems can automate processes that would otherwise consume countless hours of human labor. While this is certainly useful to most researchers, the truly exciting aspects of these techniques come from their potential to generate insights outside of human capabilities.

## 11. Potential Insights into Cognitive Processes and Clinical Manifestations

While we will not know exactly what insights these methods have to offer until they are implemented more widely, we can imagine what kinds of advancements can be built upon both advancing technology and greater understanding of the cognitive processes behind the BDT. In this section, we provide examples of how the technological advances in BDT data collection, scoring, and analysis could provide new insights regarding psychological processes. We envision a more informed approach to the collection and analysis of data from the BDT that can provide new insights into psychological processes and clinical disorders. Researchers will no longer need to rely solely on broad composite scores such as overall accuracy and time. The new technologies will facilitate more nuanced recording and analysis of construction data. BDT smart technologies in combination with machine learning techniques offer several clinical applications. For example, researchers may use these technologies to test different theories of BDT performance and gain new data that have previously been difficult or impossible to collect or analyze. These data have the potential to both answer and raise questions about *how* and *why* various factors can influence performance, including both clinical disorders and normal variations in spatial abilities.

To illustrate how these technologies may contribute to cognitive theories on individual differences, we provide a high-level overview of one of the strongest clinical application areas for BDT research: autism spectrum disorder (ASD).

### Understanding Differences in BDT Performance among Those Diagnosed with Autism Spectrum Disorder (ASD)

For decades, researchers have observed that individuals diagnosed with ASD perform differently than those without this diagnosis on the BDT (e.g., Bölte et al. 2008). The direction and magnitude of these differences depends in part on what information is reported. In some cases, the difference is reported as an overall *higher* score for individuals with ASD, but this advantage is often offset by an increase in the time needed to complete the tasks. One explanation for these differences is weak central coherence (*WCC*) theory (Happé and Frith 2006), which suggests that individuals with ASD perform well on tasks that focus on local, individual aspects of the task, but relatively poorly when the task requires integrating concepts as a whole (i.e., the “Gestalt”). Whether these cognitive biases lead to enhanced, reduced, or comparable performance to control groups depends on the specific target designs used.

Existing research on the spatial abilities of individuals with ASD raises some important questions that new BDT technologies could help to address. We know very little about the actual *performance* of individuals with ASD, particularly the specific building strategies these individuals use. Gaining such information would corroborate the findings of previous studies as well as shed new light on performance at a more fine-grained level. Researchers could record and analyze constructions at all stages. For example, researchers could record whether participants diagnosed with ASD are more likely to be affected by the placement of previous blocks, even at the expense of building the overall figure. Knowing this sort of information could allow us to have a much more precise and informed evaluation of weak central coherence theory or alternative theories.

Second, the use of smart technologies in the BDT could also shed light on heterogeneity in performance. Currently, most research on the BDT focuses on group differences (e.g., ASD versus controls, or children of different ages). However, there may be substantial heterogeneity within groups. By focusing only on inference from experimental design (and not individual constructions), we may lose information that could be vitally important to understanding the processes that are involved, how they may develop, and how different disorders may affect these processes. For example, there may be broad differences between ASD individuals versus controls that can be captured by traditional scoring methods; however, there may be substantial differences between different ASD individuals in exact block placing sequences or the time taken for each individual action.

## 12. Addressing Limitations of These Methods and Techniques

Although these new technologies are exciting and have the potential to transform both the use and score of the BDT, it is also important to consider some of the limitations. The technologies that we describe here are still emerging, and many of the limitations stem from their newness.

One obvious limitation is cost; these technologies are currently expensive. However, we have every reason to believe that these technologies will become less expensive as they become more commonly used. The cost is likely to decrease exponentially as the technology improves and becomes more widely produced and distributed. One analogous example is the cost of eye tracking equipment, which, in some cases, has exceeded USD 50,000. Over time, less expensive (but still effective) systems have become available, and researchers can now obtain reasonably good eye tracking data for a fraction of the initial cost. Consequently, eye tracking is becoming increasingly popular.

Further, we are only beginning to understand the extent and kinds of insights that can be made using these methods. However, technological versions of BDT have already begun to allow for insights into individual differences, which can be the beginning for understanding cognitive processes (Cha et al. 2020; Rozencwajg and Corroyer 2001). Likewise, we are just beginning to learn what insights unsupervised machine learning can provide. Unsupervised machine learning is a bottom-up process. Thus, this method has the potential to find patterns in individual’s building data across multiple variables, including both block placements and time, to find commonalities and differences across groups, individuals, and even within individuals. Additionally, psychologists and cognitive scientists would still have the job of deriving meaning from the patterns found by unsupervised machine learning. Thus, not only do these methods serve as a tool for researchers to measure and record more during the administration of the BDT, but they can also serve as a catalyst for deeper thinking and possible discovery by human scientists.

## 13. Future Directions: Building upon These Methods and Techniques

Currently, these methods still have unexplored potential as a valuable tool for researchers and clinicians. Given the overall increase in interest and use of machine learning methods for science in all fields, the time is ripe to investigate how these technologies can be used to support psychological research. Before these methods can become widespread, there needs to be support and further work on their implementation. Specifically, studies with a large number of participants should be conducted using both the collection and analysis methods outlined in this paper to demonstrate what kinds of patterns in building processes could be found at a large scale. Further, more specific variables within the building process, such as errors or time for each action, could be evaluated separately, but also on a much larger scale than previous studies.

### The Potential Use of These Technologies for Clinical Practice

Each of the measurement and analytical methods outlined above can provide insight into how individuals complete the BDT and can be used for the discovery of important individual differences. The combination of these insights and advanced technology can be used to advance the use of the BDT. In particular, the potential for real-time assessment can significantly change the way that the BDT is typically administered. Researchers and clinicians could have individuals complete the BDT with a set of embedded sensor system blocks, such as the sCubes systems, and have real-time data that could be automatically scored (McKee et al. 2023). Such automated scoring systems have started to be used for other cognitive tests; for example, recent work has created a machine learning system for automatically scoring the Rey–Osterrieth Complex Figure Test (Simfukwe et al. 2021). Further, any insights that come from a large BDT dataset analyzed with unsupervised clustering machine learning could be incorporated into real-time assessment as well. Machine learning systems could be designed to detect select strategies or building characteristics while an individual is completing the test. From a clinical perspective, automated scoring offers several advantages. For example, it reduces the cognitive load of administering the task so the examiner may attend to other important aspects of the assessment and yields richer data on which to base clinical interpretations and recommendations (Young et al. 2022). Moreover, the advanced process scores that technology-enabled versions of block design can provide may offer new insights into the specific brain networks involved in spatial weaknesses and how those impairments may manifest as functional outcomes. Automated systems do not eliminate the need for a human examiner; rather, they allow examiners to focus on tasks that are uniquely human, such as behavioral observations, building rapport, and testing the limits of an examinees’ abilities.

## 14. Conclusions

The BDT is an important neuropsychological test with diverse research, educational, and clinical applications. Traditional versions of the BDT offer the advantages of tangible manipulatives in a physical assembly-based task yet are limited by a human examiner’s ability to record nuanced process information in real-time. Advanced technologies enhance the measurement capabilities of the BDT by easily recording minute details about how individuals complete this task (Table 1). Further, artificial intelligence techniques open the door for analyzing data from the BDT in ways not previously possible. Most of these technologies are still relatively new, and more research and development is needed. We encourage the further investigation and usage of these technologies for the BDT as well as for other neuropsychological assessments. The general methodologies and technologies reviewed in this paper could be widely applied to other tests of cognitive abilities and may deepen our understanding of cognitive functions through a fine-grained examination of behaviors and should be explored further.

## Figures and Tables

**Table 1:** Summary of methodologies used for advancing measurement in the block design test.

Block Design Variable	Description	Tool
Fixation Length and Sequence	Frequency of individual looking at different components of the block design test and sequence of where the individual looks.	Corneal Imaging
		VR-Based Eye-Tracking
Sequenced Individual Block Placements	Detailed record of each block placement in order, including erroneous placements.	Computerized Administration
		Virtual Reality Administration
		Embedded Sensor System
		Computer Vision with Video Recordings
Pattern identification in block placements across individuals	Analysis of block sequence data to find clusters of data, such as categories of different strategies	Supervised Machine Learning
		Unsupervised Machine Learning

## Data Availability

No new data were created or analyzed in this study. Data sharing is not applicable to this article.
